# Comparison of gamma index based on dosimetric error and clinically relevant dose–volume index based on three-dimensional dose prediction in breast intensity-modulated radiation therapy

**DOI:** 10.1186/s13014-019-1233-0

**Published:** 2019-02-26

**Authors:** Akari Kaneko, Iori Sumida, Hirokazu Mizuno, Fumiaki Isohashi, Osamu Suzuki, Yuji Seo, Keisuke Otani, Keisuke Tamari, Kazuhiko Ogawa

**Affiliations:** 1 Department of Radiation Oncology, Osaka University Graduate School of Medicine, 2-2 Yamada-oka, Suita, 565-0871 Osaka Japan; 2 Department of Radiology, Suita Tokushukai Hospital, 21-1 Senrioka-nishi, Suita, 565-0814 Osaka Japan

## Abstract

**Background:**

Measurement-guided dose reconstruction has lately attracted significant attention because it can predict the delivered patient dose distribution. Although the treatment planning system (TPS) uses sophisticated algorithm to calculate the dose distribution, the calculation accuracy depends on the particular TPS used. This study aimed to investigate the relationship between the gamma passing rate (GPR) and the clinically relevant dose–volume index based on the predicted 3D patient dose distribution derived from two TPSs (XiO, RayStation).

**Methods:**

Twenty-one breast intensity-modulated radiation therapy plans were inversely optimized using XiO. With the same plans, both TPSs calculated the planned dose distribution. We conducted per-beam measurements on the coronal plane using a 2D array detector and analyzed the difference in 2D GPRs between the measured and planned doses by commercial software. Using in-house software, we calculated the predicted 3D patient dose distribution and derived the predicted 3D GPR, the predicted per-organ 3D GPR, and the predicted clinically relevant dose–volume indices [dose–volume histogram metrics and the value of the tumor-control probability/normal tissue complication probability of the planning target volume and organs at risk]. The results derived from XiO were compared with those from RayStation.

**Results:**

While the mean 2D GPRs derived from both TPSs were 98.1% (XiO) and 100% (RayStation), the mean predicted 3D GPRs of ipsilateral lung (73.3% [XiO] and 85.9% [RayStation]; *p* < 0.001) had no correlation with 2D GPRs under the 3% global/3 mm criterion. Besides, this significant difference in terms of referenced TPS between XiO and RayStation could be explained by the fact that the error of predicted V_5Gy_ of ipsilateral lung derived from XiO (29.6%) was significantly larger than that derived from RayStation (− 0.2%; *p* < 0.001).

**Conclusions:**

GPR is useful as a patient quality assurance to detect dosimetric errors; however, it does not necessarily contain detailed information on errors. Using the predicted clinically relevant dose–volume indices, the clinical interpretation of dosimetric errors can be obtained. We conclude that a clinically relevant dose–volume index based on the predicted 3D patient dose distribution could add to the clinical and biological considerations in the GPR, if we can guarantee the dose calculation accuracy of referenced TPS.

## Background

Patient-specific quality assurance (QA) is essential in intensity-modulated radiation therapy (IMRT). No current treatment planning system (TPS) completely considers dose uncertainties due to the beam-delivery system in IMRT, for example, source distribution, leaf thickness of multileaf collimators (MLCs), leakage, tongue-and-groove structures, and the dosimetric effective MLC offset [1]. The gamma passing rate (GPR), introduced by Low et al. [2], conveniently combines the dose difference and the distance to agreement by considering the difference between the planned and measured dose distributions. The GPR, which is widely used for patient-specific QA, is recommended by the American Association of Physicists in Medicine Task Group (AAPM TG) 119 [3] and 218 [4]. Although conflicting findings about the effectiveness of the GPR regarding error detection have often been reported [5–7], the GPR still provides evidence of the dose error between planned and delivered dose distributions that is sufficiently strong to lead to the approval of clinical treatments [3, 4].

Measurement-guided dose reconstruction (MGDR) [7–9] using commercial 3DVH software (Sun Nuclear Corporation, Melbourne, FL, USA) [10] has been used to predict the delivered 3D dose distribution, including potential dose error during beam delivery. On the basis of the differences between the predicted and planned distributions, various clinically relevant dose–volume indices have been discussed, such as per-organ dose–volume histogram (DVH) metrics, tumor-control probability/normal tissue complication probability (TCP/NTCP) [11]. With regard to the relationship between GPR and the predicted DVH metrics, Nelms et al. [7] demonstrated that the GPR is not correlated with dose errors in organs at risk (OARs).

Most previous studies of MGDR for QA used commercial software, such as COMPASS (IBA Dosimetry, Schwarzenbruck, Germany) [12] and 3DVH. These systems require additional beam modeling or re-calculation of the dose distribution, which generates extra uncertainty in addition to the uncertainty of dose calculation of the original plan. To overcome this issue, we proposed a simplified MGDR procedure that uses the in-house software developed in our previous work [13–16], which only adds a local error distribution along with the incident photon flux passing through the beam’s eye view.

In addition, several studies of MGDR [11, 17] have demonstrated that the results of the predicted DVH metrics or TCP/NTCP change depending on machine delivery errors and TPS beam modeling. Although the current methods to calculate the TPS dose distribution are sophisticated, elements constituting the beam model and the type and number of modeling parameters are not the same for each TPS. For example, the XiO TPS (ELEKTA Instrument AB, Stockholm, Sweden) uses a single-photon-source model [18], whereas the RayStation TPS (RaySearch Medical Laboratories AB, Stockholm, Sweden) uses a dual-source photon-beam model [19], which comprises a primary-photon-source target and an additional component serving as an extra-focal photon source (e.g., the primary collimator and flattening filter) [20, 21]. Furthermore, although XiO has a few beam modeling parameters that users can optimize, RayStation has many [22–24].

Radiation pneumonitis has been indicated after irradiation for breast-cancer radiotherapy [25]. In breast IMRT, the dose absorbed in the lung should be considered because the mean dose in the lung for breast IMRT typically exceeds that for three-dimensional conformal radiotherapy [26]. Because the TCP/NTCP values are associated with the TPS-calculated doses, several studies have reported that calculated NTCP values for the lung, combined with the inhomogeneity corrections for dose calculations, are sensitive to differences in the calculation algorithms [27, 28].

The present study aims to verify the usefulness of the predicted clinically relevant dose–volume index (DVH metrics and TCP/NTCP values) based on the predicted 3D patient dose distribution by comparison with the 2D GPR, the predicted 3D GPR, and the predicted per-organ 3D GPR using the in-house MGDR software in breast IMRT. In addition, the issue that indices derived from the predicted 3D patient dose distribution change significantly depending on the accuracy of the referenced TPS is also demonstrated.

## Methods

### Treatment planning and delivery

This study is based on 21 clinically approved left-sided breast IMRT plans, whose parameters are summarized in Table 1. All patients were treated with an ARTISTE (Siemens Medical Systems, Concord, USA) using a 6-MV photon beam and 160 MLCs with a 5-mm leaf width. Computed tomography (CT) image acquisition and radiation treatment were performed under free breathing. All IMRT plans involved two tangentially opposed beams in fixed-gantry step-and-shoot delivery using the XiO TPS. Gantry and collimator angles were selected to avoid contralateral breast irradiation and minimize exposure to the ipsilateral lung. The prescription dose was 50 Gy in 25 fractions to D_50%_ of the planning target volume (PTV). The PTV and OARs were contoured, and the dose constraints were assigned based on the protocol of the Radiation Therapy Oncology Group 1005 [29]. The first 5 mm of tissue under the skin was omitted from the PTV. Based on target prescription and dose–volume constraints, IMRT plans were inversely optimized in XiO.

**Table 1:** Description of 21 plans

	Value (range)
# Beams	2 (all plans)
Minimum segment size	2 × 2 cm^2^ (all plans)
Minimum monitor units (MUs) per segment	5 MU (all plans)
Mean (range) # segments	35 ± 7 (19–46)
Mean (range) equivalent square side (cm)	8.6 ± 0.8 (7.4–10.2)
Dose–volume constraint of PTV: D_95%_	≥95% (47.5 Gy)
Dose–volume constraint of ipsi. lung: V_20Gy_	< 15% (per protcol), < 20% (variation acceptable)
Dose–volume constraint of heart: D_mean_	< 4 Gy

D_95%_: dose coverage 95% volume, ipsi.: ipsilateral, V_20Gy_: volume receiving at least 20 Gy, D_mean_: dose coverage mean volume

The Digital Imaging and Communications in Medicine-Radiotherapy (DICOM-RT) CT image, plan, and structure files of all 21 breast IMRT plans were exported from XiO to RayStation. The delivery monitor unit and shape of segments were exactly the same for all fields for each TPS plan. Both TPSs used a superposition algorithm with a 2-mm grid size to calculate the dose distribution. Note that XiO and RayStation were approved by commissioning tests defined by AAPM TG 53 [30] and ESTRO Booklet 7 [31] and are used in our institution of clinical radiotherapy.

### Patient-specific QA based on dosimetric error

For patient-specific QA based on dosimetric error, 2D GPRs were calculated from the measured and planned dose distributions obtained by each TPS. Per-beam measurements were performed for all plans on the coronal plane (depth 5 cm, source-to-axial distance 100 cm) and at zero gantry angle using MapCHECK (Sun Nuclear Corporation, Melbourne, FL, USA), of which spatial resolution was 5-mm with a detector density. 2D GPRs were analyzed by the commercially supplied software included with MapCHECK with the criteria of 3% global/3 mm, 3% global/2 mm, and 2% global/2 mm with a lower threshold of 10%. We adopted multiple gamma criteria in this study to understand the cause and impact of error by comparing with multiple sensitivity as mentioned in AAPM TG 218 [4].

### Patient-specific QA based on predicted 3D patient dose distribution

For patient-specific QA based on the predicted 3D patient dose distribution, we calculated the predicted 3D GPR, the predicted per-organ 3D GPR, and the predicted clinically relevant dose–volume indices (DVH metrics and the value of TCP/NTCP of the PTV and OARs) using the in-house MGDR software application [14].

**Fig. 1:**
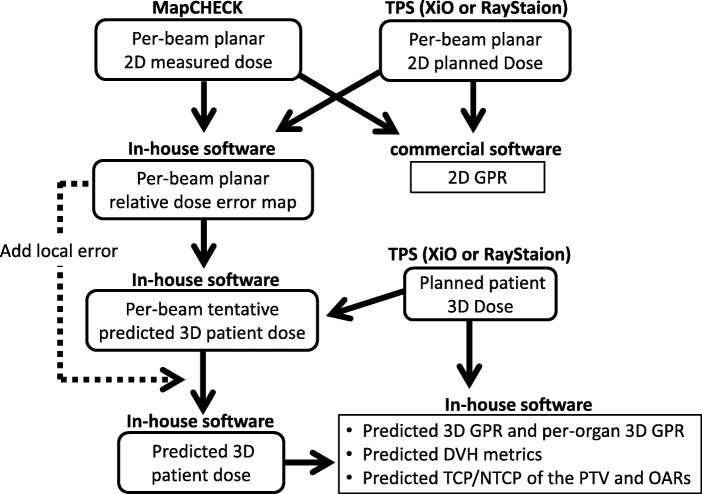
The procedure for deriving the predicted 3D GPR, the predicted per-organ 3D GPR, and the predicted clinically relevant dose–volume indices (DVH metrics and the value of TCP/NTCP of the PTV and OARs)

**Fig. 2:**
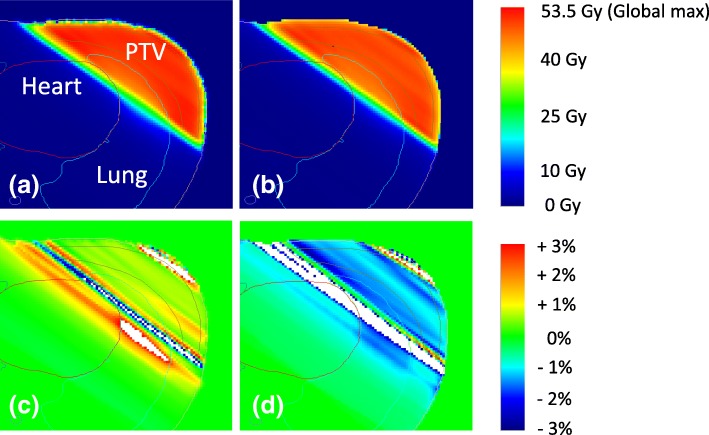
Predicted dose and dose-difference distributions on a typical axial plane. (**a**) The predicted dose distribution planes derived from XiO TPS planned dose and (**b**) RayStation TPS planned dose. Dose-difference distribution (predicted versus planned dose) obtained by (**c**) XiO and (**d**) RayStation

The procedure for deriving these indices is shown in Fig. 1. First, we created a per-beam planar relative dose error map with a 5-mm grid resolution, which is based on the per-beam planned 2D dose by each TPS and the measured 2D dose by MapCHECK. Second, the per-beam tentative predicted 3D patient dose distribution was reconstructed from the per-beam planar relative dose error map and the planned patient 3D dose distribution by each TPS with a 2-mm calculation grid. Third, the ray from the source to each tentative 3D dose grid was defined. The local error of intersection point between the ray and the per-beam planar relative dose error map located on the isocenter plane was obtained by the linear interpolation. Thus, the per-beam tentative predicted 3D dose grid was given the local error along the ray. Fourth, the predicted 3D patient dose distribution was obtained by summation of the per-beam tentative predicted 3D dose grid [14]. Typical axial planes of the predicted 3D patient dose distribution and dose-difference distributions with the planned 3D dose distribution are shown in Fig. 2.

Finally, we calculated the predicted 3D GPR and the predicted per-organ 3D GPR with criteria of 3% global/3 mm, 3% global/2 mm, and 2% global/2 mm with a lower threshold of 3% and the predicted clinically relevant dose–volume indices by comparison with the predicted 3D dose distribution and the planned patient 3D dose distribution of each TPS. The results derived from the XiO TPS were compared with those from the RayStation TPS.

The TCP is calculated using Niemierko’s EUD-based model [32]:

$$ \mathrm{TCP}\left({D}_i\right)=\frac{1}{1+{\left(\frac{TC{D}_{50}}{D_i}\right)}^{4{\gamma}_{50}}}, $$

where D_*i*_ is the original or predicted dose of the *i*-th voxel, TCD_50_ is the dose required for a TCP of 50%, and γ_50_ is the slope of the normalized tumor dose-response curve at TCD_50_.

The NTCP is calculated using the relative seriality model [33, 34]:

$$ \mathrm{NTCP}={\left[1-\prod \limits_{i=1}^M{\left[1-P{\left({D}_i\right)}^s\right]}^{\Delta {v}_i}\right]}^{\frac{1}{s}},\kern0ex \mathrm{where}\kern0.35em P\left({D}_i\right)={2}^{-\exp \left[ e\gamma \left(1-\left({D}_i/{D}_{50}\right)\right)\right]}. $$

The *P*(*D*_*i*_) is the probability of no cell surviving, and *s* is the generalized parameter describing relative seriality. The fractional volume, Δ*v*_*i*_, at doses *D*_*i*_ is most conveniently obtained from the *M* bins of the differential DVH for the organ in question. D_50_ is the total dose required for a NTCP of 50%, and γ is the slope of the maximum normalized normal tissue dose-response curve. Table 2 summarizes the resource parameters for calculating the TCP/NTCP.

**Table 2:** Parameters for calculating TCP/NTCP

Structure	Endpoint	Tissue-specific parameter	TCD_50_/D_50_ (Gy)	γ_50_/γ	s	Ref.
PTV	Local control	−7.2	30.89	1.3		[37, 38]
Lung	Pneumonitis	1	26.16	0.973	0.012	[28]
Heart	Late cardiac mortality	3	52.3	1.28	1	[39]

TCD_50_: dose required for 50% probability of tumor control, D_50_: dose required for 50% probability of normal tissue complication, γ_50_: slope of normalized tumor dose-response curve at TCD_50_, γ: slope of maxixum normalized normal tissue dose-response curve, s: relative seriality parameter

### Statistical analysis

The normal distribution of the data was determined using the Shapiro–Wilk test. Depending on the results, data were compared using the two-tailed paired *t*-test or the Wilcoxon signed-rank test, and the correlation was determined using Pearson’s or Spearman’s correlation coefficient, R. A *p* value < 0.05 was considered statistically significant. All statistical analyses were performed using the software R (The R Foundation for Statistical Computing, Vienna, Austria).

## Results

### 2D GPR and predicted 3D GPR

The top of Table 3 shows the mean 2D GPRs obtained by per-beam planar dose analyses. Regarding the clinical decision, because all plans were planned by TPS XiO, these were verified with the XiO planned dose and accepted with criteria of 2D GPRs 95% or over under 3%/3 mm criterion. A few plans in which 2D GPRs were less than 95% were considered clinically acceptable. The mean 2D GPRs from RayStation were 100% under the 3%/3 mm criterion, which were significantly higher (*p* < 0.001) than those from XiO (98.1%). Under the 3%/2 mm and 2%/2 mm criteria, a similar tendency was also seen.

**Table 3:** Summary of the GPR results

	XiO	RayStation	
	Mean ± SD (range)	Mean ± SD (range)	*P* value
2D GPR			
3%/3 mm	98.1 ± 1.9	100.0 ± 0.1	*p* < 0.001
	(93.5–100)	(99.5–100)	
3%/2 mm	95.2 ± 2.9	99.8 ± 0.5	*p* < 0.001
	(86.5–99.0)	(97.7–100)	
2%/2 mm	93.3 ± 3.7	99.5 ± 0.8	*p* < 0.001
	(82.4–98.7)	(95.7–100)	
predicted 3D GPR			
3%/3 mm	91.3 ± 1.6	93.9 ± 1.0	*p* < 0.001
	(88.6–94.5)	(91.8–96.2)	
3%/2 mm	85.9 ± 2.3	91.2 ± 1.1	*p* < 0.001
	(82.2–91.0)	(89.0–93.5)	
2%/2 mm	82.1 ± 3.5	89.5 ± 1.8	*p* < 0.001
	(77.2–87.8)	(86.1–92.3)	

2D GPR averaged for all applicable fields. The predicted 3D GPR derived from each TPS (XiO or RayStaion)

The bottom of Table 3 shows the predicted 3D GPRs. Under all criteria, the mean predicted 3D GPRs for RayStation were significantly higher (*p* < 0.001) than those for XiO. In addition, each predicted 3D GPR was lower than 2D GPR under all criteria. For example, the predicted 3D GPRs for XiO under 3%/3 mm were lower than the 2D GPRs for XiO under 3%/3 mm.

### The predicted per-organ 3D GPR

**Fig. 3:**
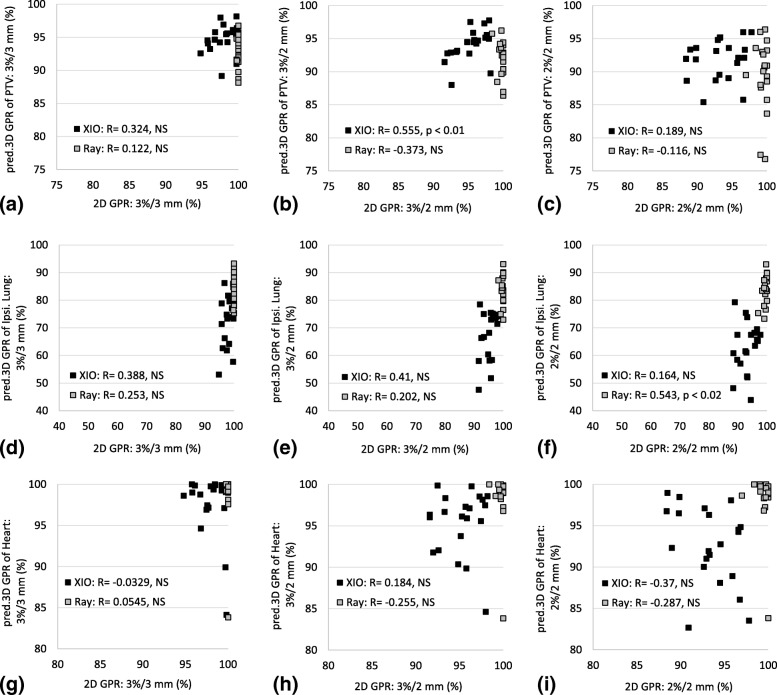
Correlation between 2D GPR and predicted per-organ 3D GPR: (**a**), (**b**), (**c**) PTV, (**d**), (**e**), (**f**) ipsilateral lung, and (**g**), (**h**), (**i**) heart. “R” denotes the Pearson correlation coefficient or Spearman Rank-Order Correlation coefficient. Pred. = Predicted. Ray = RayStation. Ipsi. = Ipsilateral. NS = not significant

Figure 3 shows the correlations between the predicted per-organ 3D GPRs and 2D GPRs. The predicted 3D GPRs of PTV, ipsilateral lung, and heart do not correlate with the 2D GPRs except two datasets (the predicted 3D GPRs of PTV under 3%/2 mm derived from XiO and the predicted 3D GPRs of ipsilateral lung under 2%/2 mm derived from RayStation). In addition, these datasets were in moderate correlation as shown in Fig. 3.

Regarding the referred difference in TPS, the mean predicted 3D GPRs of ipsilateral lung derived from RayStation was significantly higher than those from XiO under all criteria (*p* < 0.001), whereas the mean predicted 3D GPRs of PTV derived from RayStation were significantly lower than those from XiO under the 3%/3 mm and 3%/2 mm criteria (*p* < 0.001) (Table 4).

**Table 4:** Summary of predicted per-organ 3D GPR derived from each TPS (XiO or RayStaion)

predicted per-organ 3D GPR	XiO	RayStation	*P* value
	Mean ± SD (range)	Mean ± SD (range)	
PTV			
3%/3 mm	94.9 ± 2.1	93.1 ± 2.4	*p* < 0.001
	(89.2–98.2)	(88.2–96.8)	
3%/2 mm	93.9 ± 2.4	91.6 ± 2.8	*p* < 0.001
	(88.0–97.8)	(86.4–96.2)	
2%/2 mm	91.9 ± 3.1	89.4 ± 5.2	Not Significant
	(85.4–96.0)	(76.8–96.4)	
Ipsilateral lung			
3%/3 mm	73.3 ± 9.1	85.9 ± 5.0	*p* < 0.001
	(53.1–86.2)	(75.4–93.3)	
3%/2 mm	67.7 ± 8.7	84.6 ± 5.4	*p* < 0.001
	(47.6–78.5)	(73.0–93.1)	
2%/2 mm	63.1 ± 8.9	84.3 ± 5.3	*p* < 0.001
	(43.9–79.3)	(73.3–93.0)	
Heart			
3%/3 mm	97.6 ± 3.9	98.8 ± 3.5	*p* < 0.05
	(84.1–100)	(83.8–100)	
3%/2 mm	95.4 ± 3.8	98.4 ± 3.5	*p* < 0.001
	(84.6–99.9)	(83.8–100)	
2%/2 mm	92.6 ± 4.8	98.4 ± 3.4	*p* < 0.001
	(82.7–99.0)	(83.8–100)	

### The predicted clinically relevant dose–volume indices

**Fig. 4:**
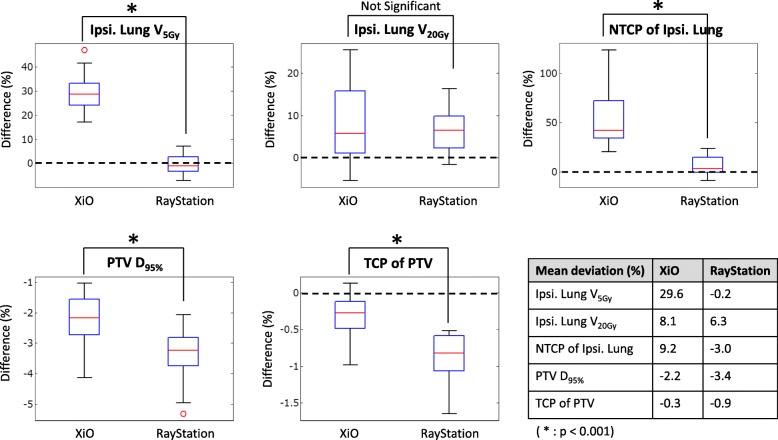
Deviations between planned and predicted DVH metrics and TCP/NTC*P* values of all plans for ipsilateral lung and PTV. Box plot shows median (red lines), 25% or 75% quartile ranges around the median (box width) and upper or lower limits (T) of each data. V_5Gy_ or V_20Gy_: volume receiving at least 5 Gy or 20 Gy of ipsilateral lung. * denotes *p* < 0.001. Ipsi. = Ipsilateral

Table 5 shows the mean predicted clinically relevant dose–volume indices. Figure 4 shows the mean deviations between the predicted and planned clinically relevant dose–volume indices. The mean deviations between the predicted and planned V_5Gy_ and NTCP of ipsilateral lung derived from XiO (V_5Gy_: 29.6%, NTCP: 9.2%) were significantly larger than those derived from RayStation (V_5Gy_: − 0.2%, NTCP: − 3.0%; *p* < 0.001), whereas the deviations between the predicted and planned V_20Gy_ of ipsilateral lung derived from RayStation and XiO showed no significant difference. Besides, the deviations between the predicted and planned PTV D_95%_ and the TCP of the PTV derived from XiO were significantly better than those derived from RayStation (*p* < 0.001).

**Table 5:** Comparison of predicted clinically relevant dose–volume indices derived from each TPS (XiO or RayStaion)

Predicted clinically relevant dose–volume indices	XiO Mean ± SD	RayStation Mean ± SD	*P* value
PTV D_95%_ (Gy)	47.0 ± 0.5	46.8 ± 0.4	*p* < 0.001
Heart D_mean_ (Gy)	2.5 ± 0.9	2.4 ± 0.9	*p* < 0.001
Lung V_5Gy_ (%)	23.7 ± 6.4	19.2 ± 5.8	*p* < 0.001
Lung V_20Gy_ (%)	10.3 ± 4.8	10.0 ± 4.6	*p* < 0.005
TCP of PTV	0.9199 ± 0.0034	0.9184 ± 0.0029	*p* < 0.005
NTCP of Ipsilateral Lung	0.0079 ± 0.0074	0.0062 ± 0.0061	*p* < 0.001
NTCP of Heart	0.0034 ± 0.0037	0.0035 ± 0.0037	Not significant

PTV D_95%_: dose coverage 95% volume of PTV. Heart D_mean_: dose coverage mean volume of Heart. Lung V_5Gy_ or V_20Gy_: volume receiving at least 5 Gy or 20 Gy of ipsilateral lung

## Discussion

This study compares the 2D GPRs, the predicted 3D GPRs, the predicted per-organ 3D GPRs, and the predicted clinically relevant dose–volume indices derived from the two TPSs. The mean 2D GPRs and the mean predicted 3D GPRs from RayStation are higher than those from XiO (Table 3). The 2D GPR would be satisfactory for patient-specific QA with regard to evaluation of the dosimetric accuracy of the planned and measured data.

However, as several researchers have pointed out, the 2D GPR does not necessarily detect clinically significant dosimetric error, such as the dose deviation of OARs [5–7]. As shown in Table 4 and Fig. 3, under 3%/3 mm criterion, the 2D GPRs do not correlate with the predicted per-organ 3D GPRs. Even under stricter criteria (i.e., 3%/2 mm or 2%/2 mm), the 2D GPRs do not necessarily correlate with the predicted per-organ 3D GPRs.

Moreover, although the predicted per-organ 3D GPRs indicated an existence of a predicted error, it is difficult to understand the influence of such an error in a clinical context. The deviation between the predicted and planned clinically relevant dose–volume indices helps in this understanding, as shown in Table 5 and Fig. 4.

In addition, the predicted 3D GPRs, the predicted per-organ 3D GPRs, and the predicted clinically relevant dose–volume indices derived from the two TPSs have significant differences (Tables 3, 4 and 5 and Figs. 3 and 4), which implies that the predicted 3D dose distribution is affected by the accuracy of the TPS model. With regard to the heart, it is difficult to ascertain the effect of the different TPSs in predicted 3D dose distributions because the relative volume of the heart is less than that of other organs in all plans.

**Fig. 5:**
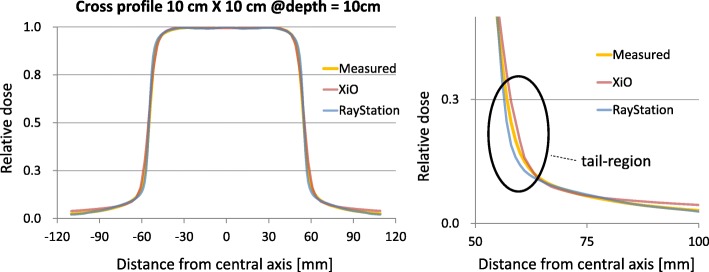
6 MV open-field cross-plane profiles measured and calculated by both TPSs for a field size of 10 cm × 10 cm and a depth of 10 cm, all at a resolution of 0.1 cm and normalized at the central beam axis

Regarding the open-beam profile (6 MV, 10 × 10 cm^2^) for both TPSs, Table 6 and Fig. 5 show the beam profile measured using a 3D water tank and the analysis results calculated by XiO and RayStation. The beam profile was divided into three regions and analyzed. The central region is the central part and the flattened area of the profile [80% or over of the central beam axis (CAX)]. The penumbra region represents the field edge with a rapid dose fall-off (between 20 and 80% of the CAX). The out-of-field region is outside the radiation field (up to 20% of the CAX). According to the Venselaar [35] formula, the percentage difference between the calculated and measured data is defined as follows:

$$ \updelta =100\%\times \kern0.5em \left({D}_{calculated}-{D}_{measured}\right)/{D}_{measured} $$

$$ {\updelta}_{out- of- field}=100\%\times \left({D}_{calculated}-{D}_{mea sured}\right)/{D}_{mea\mathrm{s} ured, CAX} $$

**Table 6:** Summary of the average deviation (%) and distance to agreement (mm) from curve-quality metrics for cross-plane profiles (averaged over all depths: *d*_max,_ 5, and 10 cm)

field size	XiO			RayStation		
	central (%)	penumbra (mm)	out-of-field (%)	central (%)	penumbra (mm)	out-of-field (%)
2 cm	4.36 ± 4.10	0.97 ± 0.58	2.75 ± 2.39	1.20 ± 1.69	0.51 ± 0.15	0.47 ± 0.92
5 cm	1.27 ± 2.03	0.95 ± 0.63	2.21 ± 2.06	0.54 ± 1.23	0.52 ± 0.36	0.49 ± 0.59
10 cm	0.37 ± 0.51	0.51 ± 0.39	1.39 ± 1.00	0.49 ± 1.11	0.61 ± 0.45	0.57 ± 0.73
20 cm	0.34 ± 0.50	0.50 ± 0.28	1.52 ± 0.89	0.34 ± 0.66	0.82 ± 0.68	0.79 ± 0.88

Square field sizes was 2, 5, 10, and 20 cm. Source-surface distance of all data was 100 cm. *d*_max_: depth of dose maximum

The out-of-field profile calculated by RayStation is more consistent with the measured profile than that calculated by XiO (Table 6). Because the lung structure of breast IMRT is mainly occupied by out-of-field dose, the predicted 3D GPRs of ipsilateral lung derived from RayStation are significantly higher than those derived from XiO (*p* < 0.001) (Table 4). Similarly, the deviations between the predicted and planned ipsilateral lung DVH metrics V_5Gy_ and NTCP derived from RayStation are significantly smaller than those from XiO (*p* < 0.001) (Fig. 4). These results should be attributable to the accuracy of the low-dose area and the out-of-field dose of RayStation, which comprises a dual-source model and is more sophisticated than XiO for optimizing beam modeling.

As for PTV, the predicted 3D GPRs of PTV derived from RayStation are significantly lower than those derived from XiO under the 3%/3 mm and 3%/2 mm criteria (*p* < 0.001) (Table 4). The deviations between the predicted and planned PTV DVH metrics D_95%_ and TCP derived from RayStation are significantly larger than those derived from XiO (*p* < 0.001) (Fig. 4). The steep gradient within the PTV region may be causally related to these deviations. Because the RayStation beam model used in our institution is optimized to a relatively small field of step-and-shoot IMRT compared with the XiO beam model, the penumbra of the RayStation beam profile was steeper than that of the XiO beam profile in the tail region (Fig. 5). The cross-plane profiles of the 10 × 10 cm^2^ field, which is close to the mean equal square of all 21 plans (8.6 × 8.6 cm^2^ field) (Table 1), shows that the XiO data are more consistent with measured data than the RayStation data on the central region and the penumbra (Table 6). However, the differences in value between the predicted PTV D_95%_ and TCP derived from RayStation and XiO are substantially small, as shown in Table 5.

Our in-house MGDR software does not require any TPS-like dose calculation engine found in commercial MGDR software such as 3DVH or COMPASS. Our software only adds the local error to the planned dose grid along with photon flux to predict the 3D dose distribution. Regarding inhomogeneity correction, our proposed MGDR method takes into consideration the accuracy attributed by the TPS calculation with the original dose grid. The dose validation of this software has been implemented [14]. Therefore, the proposed method is free from errors associated with incorrect recalculations and additional uncertainties.

Note that there are some limitations associated with this study. First, we do not consider the motion-interplay effect. Our previous study [15] showed that motion-interplay results in underdosing to the target. However, because the motion-interplay effect is independent of the calculation process of GPR and MGDR, it is not subject to discussion.

Second, the beam model used for the TPS to calculate and measure data can still be optimized. Table 7 shows the effects of change in leaf tip width, which is one of the parameters of RayStation beam modeling. Chen et al. stated that leaf tip width specifies “the dimension of leaf end which has a transmission factor equal to the square root of average MLC radiation transmission” [23]. Similar to the comparison of the two types of TPSs, the change of beam modeling parameter affects the 2D GPR, the predicted 3D GPR, and the predicted per-organ 3D GPR. Nonetheless, because this study was established to compare GPRs and the predicted clinically relevant dose–volume indices and the difference of indices of the predicted 3D patient dose distribution derived from the two TPSs, this issue lies outside the scope of this paper.

**Table 7:** Summary of the 2D GPR, predicted 3D GPR, and predicted per-organ 3D GPR under 2% global/2 mm criterion in 7 patients in the case of the change of beam modeling parameter of RayStation

RayStation (*n* = 7) 2% global/2 mm	Original leaf tip width: 0.1	leaf tip width: 0.25	leaf tip width: 0.4
	Mean ± SD (range)	Mean ± SD (range)	Mean ± SD (range)
2D GPR	99.8 ± 0.4	99.5 ± 0.7	98.8 ± 1.8
	(98.7–100.0)	(97.9–100.0)	(93.2–100.0)
Predicted 3D GPR	88.9 ± 1.5	88.4 ± 2.4	87.4 ± 2.9
	(86.1–90.6)	(84.7–90.5)	(83.1–90.3)
Predicted 3D GPR of PTV	88.8 ± 5.6	85.2 ± 7.0	80.6 ± 8.1
	(76.8–93.2)	(71.3–93.6)	(64.7–87.8)
Predicted 3D GPR of ipsilateral lung	85.7 ± 5.2	84.8 ± 5.8	85.7 ± 5.9
	(76.6–93.0)	(75.9–93.1)	(75.3–93.9)
Predicted 3D GPR of heart	96.8 ± 5.8	97.0 ± 4.9	97.4 ± 4.1
	(83.8–99.8)	(86.2–99.7)	(88.5–99.7)

Recently, the AAPM Medical Physics Practice Guidelines 5.a. recommended “2%/2 mm, no pass rate tolerance, but areas that do not pass need to be investigated” for the commissioning of IMRT/VMAT dose validation systems in the case of planar or volumetric arrays [36]. In addition, the AAPM TG 218 recommended, “If the plan fails, evaluate the gamma failure distribution and determine if the failed points lie in regions where the dose differences are clinically irrelevant” for gamma analysis [4]. These statements imply the importance not only of increasing the value of GPR but also of discussing where the error is located and what it clinically implies. As described in this paper, the clinically relevant dose–volume indices predicted by MGDR help to understand the effect of dose error clinically on each organ. In addition, when we interpret the predicted 3D patient dose distribution, we should be more aware of the possibility that the results depend on the accuracy of TPS dose calculation.

## Conclusions

In 21 cases of fixed-gantry step-and-shoot breast IMRT, the same DICOM plan was calculated using two different TPSs: XiO and RayStation. Using in-house software, each predicted 3D dose distribution was derived by referencing each TPS and the measured dose using a planar array detector. Although the mean 2D GPRs calculated by commercial software were 95% or over under 3% global/3 mm criterion, the predicted per-organ 3D GPRs did not correlate with the 2D GPRs under 3% global/3 mm criterion. In addition, the predicted 3D GPRs of ipsilateral lung derived from RayStation are significantly higher than those derived from XiO (*p* < 0.001) under all criteria. The clinical interpretation of these results was explained by the predicted clinically relevant dose–volume indices. The predicted ipsilateral lung DVH metrics V_5Gy_ and NTCP derived from RayStation were better than those derived from XiO because RayStation has better accuracy regarding calculation of the out-of-field and low-dose-area distribution.

Although we can assume that the GPR can detect dosimetric error for patient-specific QA, in case we require additional clinical and biological consideration of the actual irradiated dose distribution, we could add the predicted clinically relevant dose–volume indices on the presupposition that the accuracy of the dose calculation of TPS is guaranteed.

## Abbreviations

AAPM TG: American Association of Physicists in Medicine Task Group
CAX: Central beam axis
CT: Computed tomography
DICOM-RT: Digital Imaging and Communications in Medicine-Radiotherapy
DVH: Dose–volume histogram
GPR: Gamma passing rate
IMRT: Intensity-modulated radiation therapy
MGDR: Measurement-guided dose reconstruction
MLC: Multileaf collimator
OAR: Organ at risk
PTV: Planning target volume
QA: Quality assurance
TCP/NTCP: Tumor-control probability/normal tissue complication probability
TPS: Treatment planning system
